# The Histone Deacetylase Activator ITSA-1 Improves the Prognosis of Cardiac Arrest Rats by Alleviating Systemic Inflammatory Responses Following Cardiopulmonary Resuscitation

**DOI:** 10.1155/mi/8156593

**Published:** 2025-03-20

**Authors:** Chenyu Zhang, Hongyan Wei, Qiang Zhang, Haohong Zhan, Yuanzheng Lu, Yujie Li, Bo Li, Wen Huang, Feng Nian, Rong Liu, Chunlin Hu, Jie Chen

**Affiliations:** ^1^Department of Emergency Medicine, The First Affiliated Hospital of Sun Yat-sen University, The 58th Zhongshan II Road, Guangzhou 510080, China; ^2^Department of Critical Care Medicine, The First Affiliated Hospital, Sun Yat-sen University, Guangzhou, China; ^3^NHC Key Laboratory of Assisted Circulation, Sun Yat-sen University, Guangzhou 510080, China; ^4^Department of Emergency Medicine, The Seventh Affiliated Hospital of Sun Yat-sen University, No.628, Zhenyuan Road, Guangming (New) Dist., Shenzhen 518107, China; ^5^Department of Emergency Medicine, Fuzhou Hospital of Traditional Chinese Medicine Affiliated to Fujian University of Traditional Chinese Medicine, Fuzhou, Fujian, China; ^6^The First Affiliated Hospital of Guangzhou Medical University, Guangzhou, China; ^7^Department of Critical Care Medicine, The Tenth Affiliated Hospital, Southern Medical University (Dongguan People's Hospital), Dongguan 523059, Province Guangdong, China

**Keywords:** cardiopulmonary resuscitation, ITSA-1, prognosis, suberoylanilide hydroxamic acid (SAHA)

## Abstract

**Objective:** To investigate whether the histone deacetylase (HDAC) activator ITSA-1 can ameliorate systemic inflammation after cardiac arrest (CA), thereby enhancing cardiac function and neurological outcomes in rats.

**Materials and Methods:** Sixty-nine healthy adult male Wistar rats were subjected to 12 min of CA induced by Vecuronium bromide. The rats were randomly assigned to five groups: normal control, sham operation, control, suberoylanilide hydroxamic acid (SAHA), and ITSA-1. The study evaluated the effects of ITSA-1 on cardiac function, survival, and neurological functions, including the neurological deficit score (NDS) at 24-, 48-, and 72-h post-return of spontaneous circulation (ROSC) and Morris water maze performance at 72 h. Additionally, levels of TNF-*α*, IL-1*β*, glial fibrillary acidic protein (GFAP), S100*β* in plasma, and TNF-*α*, IL-1*β* in the hippocampus were measured 4 h post-ROSC. Western blot analysis was used to assess HDACs, nuclear factor kappa B (NF-*κ*B), p-NF-*κ*B, caspase-3, cleaved caspase-3, Bcl-2, and Bax protein expressions.

**Results:** ITSA-1 reduced basic life support (BLS) duration and adrenaline dosage during cardiopulmonary resuscitation (CPR) and improved cardiac and neural functions, enhancing survival compared to the control and SAHA groups. ITSA-1 decreased serum levels of IL-1*β*, TNF-*α*, GFAP, S100*β*, and hippocampal TNF-*α*, IL-1*β*, promoting neuronal survival in the CA1 region. It also inhibited glial cell activation and reduced histone acetylation, blocking the NF-*κ*B pathway and neuronal apoptosis.

**Conclusion:** ITSA-1 enhances the recovery and survival of post-ROSC rats by diminishing histone acetylation and mitigating systemic inflammation. This effect is possibly due to the inhibition of glial cell activation, increased neuronal survival in the brain, and improved cardiac output (CO) and ejection fraction (EF).

## 1. Introduction

Sudden cardiac death (SCD) is a disease characterized by high incidence and mortality rates worldwide. Approximately 550,000 people in China [1] and 350,000 in Europe [2] suffer from cardiac arrest (CA) annually, making CA a significant public health concern [3]. Despite advances in cardiopulmonary resuscitation (CPR) technology and improvements in the survival chain, the overall survival rate post-CA remains under 10%. Most patients succumb to post-CA syndrome following the return of spontaneous circulation (ROSC), primarily due to heart and brain injuries [4, 5]. Post-CA syndrome is marked by elevated circulating cytokines and adhesion molecules, the presence of plasma endotoxin, and an imbalance in leukocyte cytokine production [5]. CA significantly increases TNF-*α* levels in the striatum [6] and hippocampus [7] and raises IL-6 and TNF-*α* levels in plasma [8, 9]. These cytokines play a role in sepsis-induced cardiac dysfunction [10, 11] and are closely linked to the prognosis of severe sepsis [12].

ITSA-1, a histone deacetylase (HDAC) activator, has been shown to prevent arterial laminar shear stress-induced endothelial cell detachment and reduce VCAM-1 expression in vascular endothelial cells (vECs), with activation of HDACs rescuing vECs from the pathological responses induced by arterial laminar shear stress [13]. ITSA-1 also attenuates histone acetylation-dependent inflammatory signaling [14]. Suberoylanilide hydroxamic acid (SAHA), a nonselective global HDAC inhibitor, has been approved by the U.S. Food and Drug Administration for the treatment of hematologic cancers. The therapeutic potential of SAHA in organ dysfunction remains a subject of ongoing debate within the scientific community; literature reveals contradictory findings: certain studies have documented significant tissue-protective properties, whereas others have reported potentially deleterious effects on organ function [15–17]. However, the effects of HDACs on global cerebral ischemia-reperfusion injury induced by CA remain unclear. Despite sporadic studies investigating HDAC inhibitors' interventional effects in certain acute injury and inflammatory conditions, the specific mechanistic research regarding CA remains largely unexplored, urgently calling for comprehensive scientific investigation. In this study, we sought to elucidate the role of histone acetylation in post-CA pathophysiology. Specifically, we examined the modulation of systemic inflammatory responses by pharmacological interventions targeting HDACs, employing either an inhibitor (SAHA) or activator (ITSA-1) in vitro. Our findings demonstrate a promising therapeutic approach for optimizing CPR outcomes through targeted epigenetic regulation.

## 2. Materials and Methods

The project was approved by the Animal Research Committee of Sun Yat-sen University (SYSU-IACUC-2021-000081), and the animal experiment protocol adhered to the National Institutes of Health's guidelines on animal ethics in research [18]. Animal care was consistent with the recommendations outlined in the guidelines for the care and use of laboratory animals formulated by the National Research Council.

### 2.1. Animal Preparation, CA Model, and CPR

Sixty-nine healthy adult male Wistar rats, weighing between 349 and 415 g, were acquired from the Experimental Animal Center of Southern Medical University. We exclusively used male rats to minimize variability arising from hormonal fluctuations, which could influence HDAC activity and cardiac function. All rats had access to food and water ad libitum, were acclimatized for 4 weeks before the experiment, and were housed in a quiet environment with a 12-h light–dark cycle both before and after the experiment. Following an overnight fast, the animals were anesthetized with an intraperitoneal injection of 3% sodium pentobarbital (Sigma, St. Louis, MO, USA) at a dose of 45 mg/kg. Once the rat was completely unconscious, a 14G tracheal tube was inserted, and mechanical ventilation was initiated using a ventilator during CPR. The right femoral artery was cannulated for arterial blood pressure monitoring, and the left femoral vein was cannulated for intravenous access, with the cannulation sites being sealed with heparin (2.5 IU/ml). An ECG monitor (PHILIPS SureSigns VM4, MA, USA) continuously recorded the ECG, blood pressure, heart rate, respiratory rate, and SPO_2_. Ventilation was provided by a Harvard Rodent Ventilator (Harvard Apparatus, Holliston, MA), with a tidal volume of 0.8 ml/100 g and a respiratory rate of 55/min, maintaining SPO_2_ above 93%. Ventilation parameters were adjusted based on arterial blood gas results to keep the PCO_2_ within the range of 35–45 mmHg.

Vecuronium bromide (0.1 mg/kg, IV) was administered to immobilize the animal's respiration during tracheal intubation. The animal's blood pressure gradually decreased due to hypoxia. CA was defined when the mean arterial pressure (MAP) fell below 30 mmHg. Wistar rats exhibit greater tolerance to hypoxia compared to other rat strains. To establish a more effective injury model, we selected a 12-min CA duration based on the results of our preliminary experiments [19]. After 12 min of CA, CPR was initiated, mechanical ventilation was resumed, and epinephrine (20 µg/kg/3 min), along with sodium bicarbonate (1.0 ml/kg), was administered intravenously. Compressions were performed using a compression device developed in our laboratory at a rate of 200 compressions/min and a depth of 1/3 of the anteroposterior diameter of the thorax. ROSC was defined by the restoration of a supraventricular rhythm and a MAP above 65 mmHg for more than 15 min. If ROSC was not achieved within 15 min after resuscitation, resuscitative efforts were discontinued.

### 2.2. Experimental Design and Grouping

All rats were randomly divided into five groups:

Normal group: Except for CA and CPR, the rats received the same treatment as the other groups until the end of the operation.

Sham group: Rats underwent mechanical ventilation while Vecuronium bromide was administered.

Control group, SAHA group, and ITSA-1 group: Rats in these groups were administered normal saline, SAHA (MedChemExpress, Shanghai, China), or ITSA-1 (MedChemExpress, Shanghai, China), respectively, at the dose of 100 mg/kg or 0.5 mg/kg via intraperitoneal injection [14, 16]. These injections were given per kilogram of body weight for three consecutive days before the asphyxiated CA surgery.

Experiment 1 (*n* = 45): Five rats each in the normal and sham groups and 10 each in the control, SAHA, and ITSA groups were used to explore the effects of the drug on cardiac function 4 h after ROSC. A total of five rats were excluded from the experiment due to complications such as postoperative pulmonary hemorrhage. Additionally, survival status and neurological function, including the neurological deficit score (NDS) at 24, 48, and 72 h after ROSC, and performance in the Morris water maze at 72 h after ROSC, were assessed.

Experiment 2 (*n* = 24): Three rats each in the normal and sham groups and five each in the control, SAHA, and ITSA groups. A total of three rats were excluded from the experiment due to complications. Rats were used to observe the effects of ITSA-1 on TNF-*α*, IL-1*β*, glial fibrillary acidic protein (GFAP), and S100*β* levels in plasma, as well as TNF-*α* and IL-1*β* levels in the hippocampus 4 h after CA-ROSC. This experiment also aimed to evaluate the effect of ITSA-1 on the infiltration of astrocytes and microglia in the hippocampus 4 h after ROSC.

Relevant Time Definitions for the Study:

Pulseless Electrical Activity (PEA) Time: The time from the injection of Vecuronium bromide to when the MAP dropped below 30 mmHg.

Asystole Time: The time from the injection of Vecuronium bromide until MAP dropped below 30 mmHg, with ECG showing asystole.

Basic Life Support (BLS) Time: The time from the start of CPR until ROSC was achieved.

**Figure 1:**
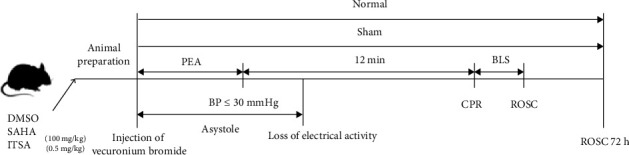
Flowchart of experiment. BLS, base life support; BP, blood pressure; CA, cardiac arrest; CPR, cardiopulmonary resuscitation; PEA, pulseless electrical activity; ROSC, return of spontaneous circulation.

The specific procedures are depicted in Figure 1.

### 2.3. Ultrasonic Measurement of Cardiac Function

About 4 h after ROSC, the rats were anesthetized by inhaling 3% isoflurane gas, and their body temperature was maintained at 37°C. A hair removal agent was used to clear the chest area of fur, and ultrasound coupling gel was applied to the surface of the rat's chest skin to enhance signal transduction. Echocardiography was performed using a Vevo-2100 high-frequency ultrasound system (VisualSonics Inc.) to evaluate left ventricular ejection fraction (LVEF) and cardiac output (CO).

### 2.4. Survival Status and Neurological Function Assessment

The primary objective of this research is to focus on the dynamic changes and improvement mechanisms of neurological function in the early stages (within 72 h) after CA. The scientific basis for selecting this time window includes the critical phase of early neural damage and repair and the peak period of inflammatory response and oxidative stress. The NDS was used to evaluate the neurological function of the rats. Independent investigators evaluated the NDS at three time points: 24, 48, and 72 h after ROSC.

### 2.5. Morris Water Maze

The Morris water maze consisted of a black metal water tank approximately 2 m in diameter and 20 cm deep, filled with opaque water at 22°C, including pool specifications (diameter of 180 cm, depth of 60 cm, water temperature maintained at 22 ± 1°C), platform details (10 cm diameter submerged 2 cm below the water surface), visual cues (description and positioning of spatial reference markers), and the video tracking system used, located in a dimly lit room. Additionally, we have outlined the training protocol in detail, covering the habituation phase (2 days of 60-s free-swimming) and the acquisition phase over 5 days (four trials per day with specific timing and randomized starting positions). We specified the assessment parameters for spatial learning evaluation (escape latency, swimming trajectory, and the proportion of time spent in the target quadrant.) The surface of the water tank was divided into four quadrants, labeled Q1, Q2, Q3, and Q4. A hidden platform was positioned 2 cm below the surface in the center of the Q3 quadrant. Each rat started from the Q2 area facing the wall and navigated to find the hidden platform in Q3. The rats were trained to locate the platform. Data were recorded using a computer system (TSE Systems GmbH, Bad Homburg, Germany) for behavioral testing, with a camera positioned at the center of the room's ceiling. The swimming path of each rat, the time taken to find the platform, the number of times the platform was crossed, the percentage of time spent in the target quadrant, and the swimming speeds of the rats in each group were analyzed. Visual cues of varying sizes and colors were placed on three walls, and a black cloth covered the tank on all four sides to conceal the researchers and computer systems. The rats were trained for three consecutive days before the CPR experiment to familiarize them with the test. After at least 3 days of recovery post-ROSC, the Morris water maze test was conducted 72 h after ROSC. The time taken to reach the designated hidden platform, the number of crossings over the hidden platform, the percentage of time spent in the target area, and the swimming speeds of rats in each group were compared.

### 2.6. Hematoxylin and Eosin (HE) Staining, Nissl Staining, and Immunohistochemical Staining

About 72 or 4 h after ROSC, the rats were euthanized under deep anesthesia. The brain was removed and bisected along the sagittal plane. Both halves were immediately immersed in a 4% paraformaldehyde solution at 4°C, then fixed, dehydrated, and embedded in paraffin before sectioning. The fixed brain tissues were cut into 5-µm-thick sections and stained using a standard HE staining kit (Servicebio, Wuhan, China). The histology of the rat brain was observed under a microscope. For Nissl staining, a Nissl staining kit (Servicebio, Wuhan, China) was used. The paraffin sections were deparaffinized and stained with a toluidine blue solution (Beyotime Institute of Biotechnology, Nantong, China). Nissl bodies were observed under an optical microscope, and in each section, Nissl-positive cells were counted in six randomly selected fields within the hippocampal CA1 area.

For immunohistochemical staining, ionized calcium-binding adapter molecule 1 (IBA-1), a 17 kDa calcium-binding protein specifically expressed in microglia of the central nervous system, and GFAP, an intermediate filament protein expressed in astrocytes, were used as markers. The sections were treated with 0.01% sodium citrate buffer for 5 min for antigen retrieval, then blocked and permeabilized in a solution of 1.5% Triton X-100/5% normal goat serum/PBS. They were incubated overnight with primary antibodies against GFAP and IBA-1 at a dilution of 1:100 (rabbit 60190, 26177, Proteintech). This was followed by incubation with a biotinylated goat anti-rabbit IgG secondary antibody for 30 min. The sections were then treated with an avidin–biotinylase complex, fixed, dehydrated, and covered with a cover glass. Five visual fields were randomly selected for observation, and Image J software was employed for quantitative analysis.

### 2.7. Measurement of Brain Injury Markers and Inflammatory Factors

The levels of IL-1*β*, TNF-*α*, GFAP, and S100*β* in serum, as well as TNF-*α* and IL-1*β* in the hippocampus, were detected using enzyme-linked immunosorbent assay (ELISA) kits (Cusabio Biotech Co. Ltd., Wuhan, China) according to the manufacturer's instructions.

### 2.8. Western Blot

About 4 h after ROSC, myocardial tissue from the anterior wall of the left ventricle of the rats was quickly excised, diced into small pieces resembling soybean granules, and stored at −80°C. Briefly, the tissue was homogenized in a protein extraction reagent containing 1 mM PMSF. The homogenate was centrifuged at 4°C and 10,000 rpm for 30 min, and the supernatant was collected. The protein concentration was determined using the BCA method. Proteins were separated by SDS–PAGE and transferred onto a PVDF membrane, which was then blocked with 5% skim milk for 2 h. The membrane was incubated overnight with primary antibodies against caspase-3, Bcl-2, Bax, and GAPDH, all diluted at 1:2000 (CST, USA). After washing with TBST, the membrane was incubated with a goat anti-rabbit antibody (diluted 1:5000 in TBST; Abcam) for 1 h at room temperature. Detection of specific bands was performed using an electrochemiluminescence western blot system. The density of immunoreactive bands was analyzed using ImageJ software (National Institutes of Health, Bethesda, Maryland, USA).

Primary antibodies used included NF-*κ*B (3033s), phosphorylated nuclear factor kappa B (NF-*κ*B) (8242S), *β*-actin (4970), PCNA (13110), GAPDH (2118), caspase-3 (9662S), cleaved caspase-3 (9661S), Bcl-2 (2870S), Bax (14796), histone H3 (9715S), HDAC1 (34589), HDAC2 (57156), HDAC3 (85057), HDAC4 (7628), HDAC6 (7612) from CST (Cell Signaling Technology, Danvers, MA, USA), and HDAC7 (ab12174), HDAC8 (ab187139), HDAC9 (ab109446), HDAC10 (ab18971), and HDAC11 (ab18973) from Abcam (Cambridge, United Kingdom).

## 3. Statistical Analysis

All statistical analyses were performed using SPSS for Windows, Version 23.0 (SPSS, Chicago, IL, USA). Data were expressed as mean ± standard deviation (SD). The comparison of means between two samples was conducted using the *t*-test, while comparisons among data from more than three groups were performed using one-way analysis of variance (ANOVA), followed by post hoc tests for multiple comparisons. Nonnormally distributed data were analyzed using nonparametric tests, with the Mann–Whitney test employed to compare the NDSs between groups. The ROSC rate was evaluated using the chi-square test. Kaplan–Meier survival analysis was used to compare survival status differences between groups. A *p*-value of less than 0.05 was considered statistically significant.

## 4. Results

### 4.1. Effects of ITSA-1 on BLS Duration and Adrenaline Dosage During CPR

The basic physiological parameters of the animals in each group and relevant parameters during CPR are detailed in Table 1. There were no significant differences in physiological parameters among the three groups. The onset time of PEA in the ITSA group was 263.5 ± 32.49 s, significantly shorter than in the control group (295.2 ± 38.36 s) and the SAHA group (301.9 ± 33.85 s). The onset time of asystole in the SAHA group was 197.40 ± 71.02 s, significantly longer than in the control group (70.90 ± 35.38 s) and the ITSA group (54.00 ± 40.06 s). The duration of BLS in the ITSA group was 145.90 ± 87.15 s, significantly shorter than in the control group (318.70 ± 231.40 s) and the SAHA group (307.90 ± 204.30 s). The dosage of adrenaline during CPR was significantly lower in the ITSA group compared to both the control and SAHA groups. The rates of ROSC were similar among the groups, with 9/12 in the control group, 8/12 in the SAHA group, and 10/12 in the ITSA group, showing no significant differences.

### 4.2. ITSA-1 Improved Cardiac Function and Survival Status of Rats After ROSC

**Figure 2:**
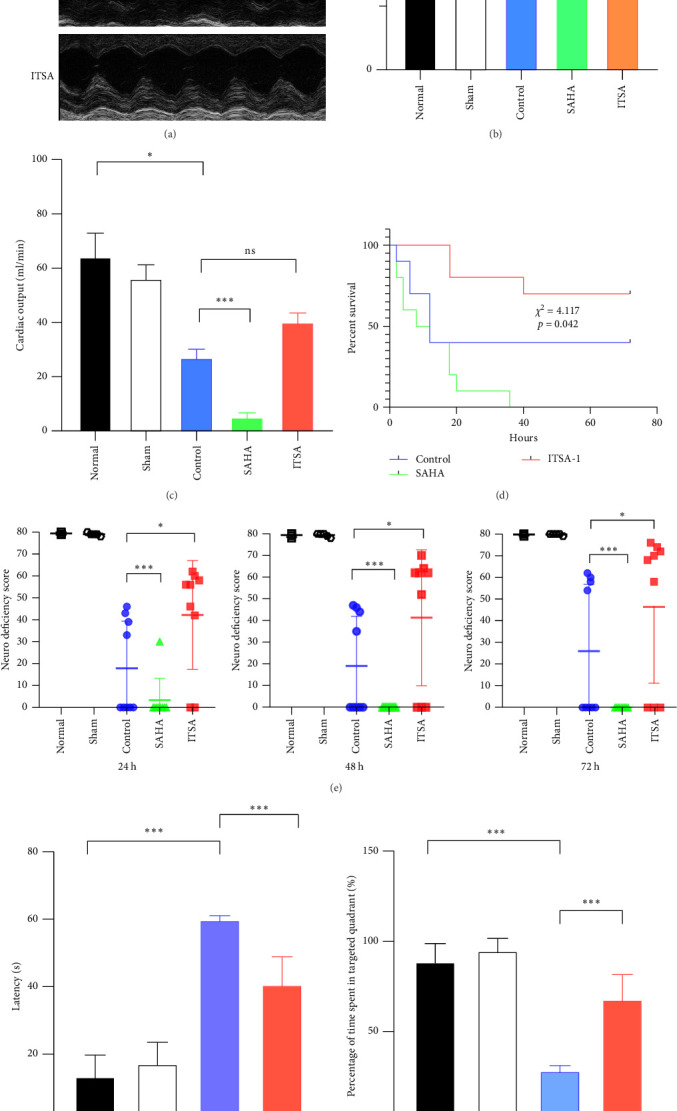
ITSA-1 improved cardiac function and survival status of rats after ROSC. (A) Representative images of echocardiography in different groups. (B) The dynamic changes of ejection fraction (EF) in different groups. (C) The dynamic changes of cardiac output (CO) in different groups. (D) Impact of HDACs activators and inhibitors on postcardiac arrest survival. Kaplan–Meier curve demonstrating improved survivals with ITSA group compared to the others (*⁣*^*∗*^*p* < 0.05). (E) Neurological deficit score of rats in each group after cardiac arrest. (F) Effect of ITSA on escape latency and percentage of time spent in targeted quadrant. Escape latency of rat in the hidden platform test, to finding the underwater platform. In the probe trail, (G) Representative swimming tracks of rat after ROSC. All data presented are means ± SEM; *N* = 5 rat per group. *⁣*^*∗*^*p* < 0.05; *⁣*^*∗∗∗*^*p* < 0.01.

Before CA, there were no significant differences in LVEF and CO among the three groups. After ROSC, the EF in the control group decreased to 61.15% ± 1.07%, in the SAHA group to 38.81% ± 9.21%, and in the ITSA group to 81.01% ± 2.88%, which was significantly higher than that in the other two groups. The CO in the ITSA group was 39.61 ± 4.12 ml/min, significantly higher than in the control group (26.49 ± 3.78 ml/min) and the SAHA group (14.49 ± 2.23 ml/min).  Figure 2 displays the Kaplan–Meier survival curves for the rats in each group following CA. No rats in the normal and sham groups died during the observation period. Within 24 h after ROSC, five rats in the control group, eight rats in the SAHA group, and two rats in the ITSA group died from refractory cardiogenic shock. Four animals in the control group and seven animals in the ITSA group survived for more than 72 h. Survival analysis indicated that ITSA-1 significantly improved the survival rate of rats 72 h post-ROSC. These results demonstrate that ITSA-1 enhanced the LV function of rats post-ROSC, thereby improving their survival rate. Conversely, SAHA appeared to worsen the LV function of rats post-ROSC, thus contributing to higher mortality rates.

### 4.3. ITSA-1 Improved Neural Function in Rats After ROSC

During the experimental observation period, no neurological deficits were observed in the normal and sham groups. Almost all rats in the SAHA group died within 24 h. At 24 h post-ROSC, the NDS for the ITSA group was 41.20 ± 7.46, which was significantly better than that of the control group (16.10 ± 6.65, *p* < 0.001). As the post-ROSC period extended, the NDS of the animals gradually improved; however, the NDS of the ITSA group remained significantly higher than that of the control group at 48 and 72 h after ROSC.

Figure 2F,G illustrates the swimming trajectory and the proportion of time spent in the target quadrant. All rats underwent spatial memory training in the water maze for three consecutive days before CA and were deemed qualified if they reached the hidden platform within 1 min. The water maze test was conducted on the third day post-ROSC. As all rats in the SAHA group died within 24 h post-ROSC, no rats from this group participated in the water maze test at 72 h post-ROSC. Results showed that rats in the normal and sham groups exhibited good spatial memory ability in the Morris water maze test, quickly finding the hidden platform even after 7 days. In contrast, CA significantly impaired the spatial memory ability of rats. In the control group, it took longer to find the target point, and they spent less time in the target area after 72 h post-ROSC. In contrast, ITSA-1 significantly reduced the memory impairment of rats post-ROSC, allowing them to stay longer in the target quadrant and reducing the time to reach the platform (escape latency: 19.7 ± 4.27 s vs. 32.50 ± 3.14 s, *p* < 0.001; percentage of time to find the platform: 58.75% ± 2.50% vs. 44.50% ± 2.89%, *p* < 0.001).

### 4.4. ITSA-1 Attenuated the Levels of IL-1*β*, TNF-*α*, GFAP, and S100*β* in Serum and TNF-*α*, IL-1*β* in the Hippocampus

**Figure 3:**
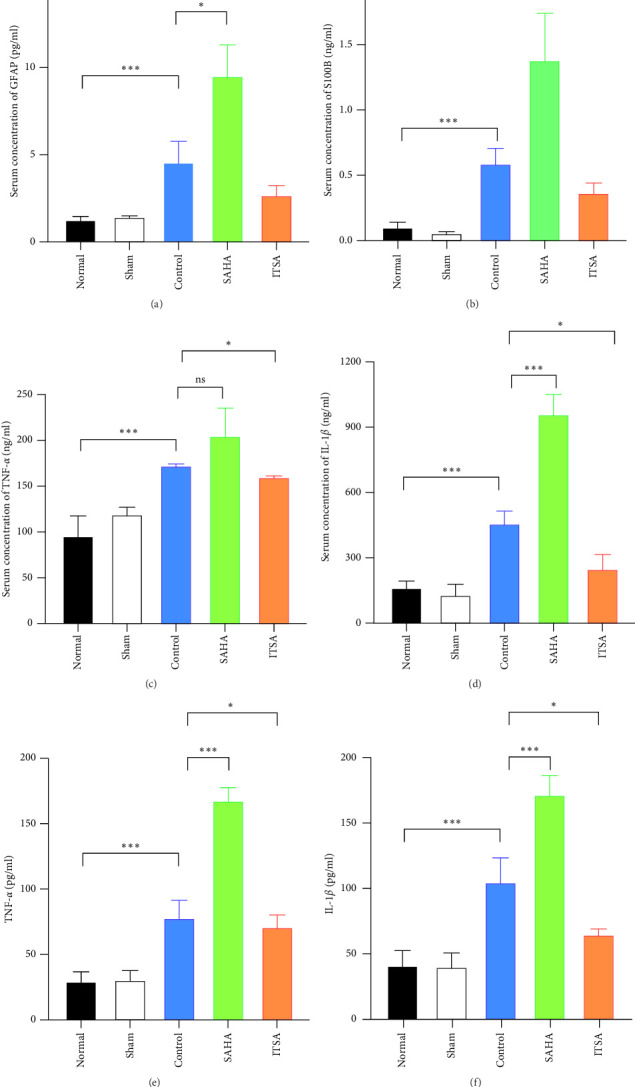
ITSA-1 Decreased the Levels of injury markers in Serum and Hippocampus. (A–D) The Serum concentration of GFAP, S100*β*, TNF-*α*, IL-1*β* are unregulated significantly after ROSC, and it was suppressed by ITSA-1 treatment instead of SAHA. (E and F) The protein concentration in hippocampus of TNF-*α*, IL-1*β* increased significantly after ROSC, and it also suppressed by ITSA-1 treatment. All data presented are means ± SEM; *N* = 5 rat per group. *⁣*^*∗*^*p* < 0.05; *⁣*^*∗∗∗*^*p* < 0.01.

Compared to the normal and sham groups, the serum brain injury markers GFAP and S100*β* in the control group were significantly increased after ROSC for 4 h (*p* < 0.05), as shown in Figure 3. The levels of GFAP and S100*β* in the SAHA group increased significantly more than those in the control and ITSA groups. The concentrations of GFAP and S100*β* were significantly lower in the ITSA group than in the control group.

In the hippocampus, the SAHA group exhibited significantly higher concentrations of TNF-*α* and IL-1*β* compared to the control and ITSA groups. Conversely, the concentrations of TNF-*α* and IL-1*β* in the ITSA group were significantly lower than those in the other two groups.

### 4.5. ITSA-1 Promoted the Survival of Neurons in the CA1 Region of Rats After ROSC

**Figure 4:**
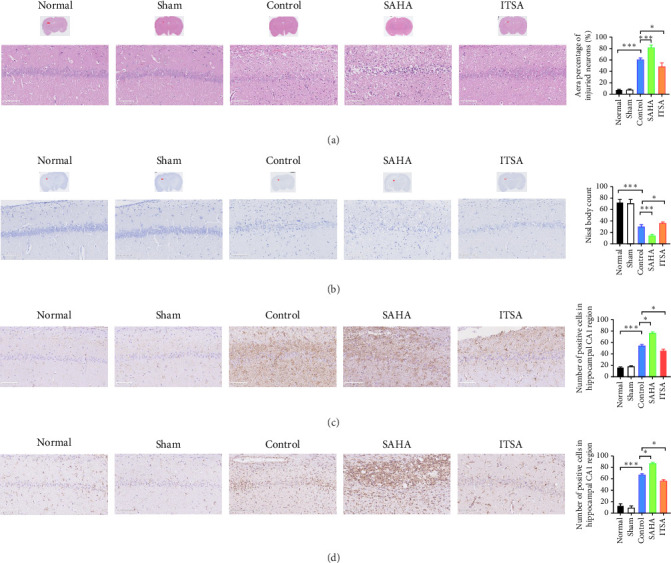
ITSA-1 Promoted the Survival of Neurons in the CA1 Region of Rats After ROSC. (A and B) HE staining and Nissl staining in the hippocampus of the rat between each group. The magnifications and scale bars were shown. (C and D) Immunohistochemical staining for GFAP, IBA-1 on hippocampus sections. Representative immunohistochemical staining show the expression of markers in the hippocampal of mice after ROSC. Original magnification = ×200, scale bar = 50 μm. *⁣*^*∗*^*p* < 0.05; *⁣*^*∗∗∗*^*p* < 0.01.

Figure 4A,B displays a representative section of the hippocampal CA1 region. The histopathology in the normal and sham groups was normal, with no neuronal necrosis observed. In the control group, some cells were disordered, and neuronal necrosis was observed. However, the morphological changes in neurons in the SAHA group were more severe than those in the control group, with a large number of neurons undergoing necrosis. The severity of neuronal necrosis in the ITSA group was less severe than that in both the control and SAHA groups.

Compared with the normal and sham groups, the number of Nissl-positive neurons in the control group decreased significantly, and the number of neurons in the SAHA group decreased even more significantly than that in the control group. The number of viable neurons in the ITSA group was significantly higher than in both the control and SAHA groups.

### 4.6. ITSA-1 Inhibits the Activation of Glial Cells in the Rat Hippocampal CA1 Region After ROSC

The results of immunohistochemical staining are depicted in Figure 4C,D. In both the normal and sham groups, the GFAP and IBA-1 positive cells were scattered throughout the CA1 area layer, displaying a resting state with small cell bodies distributed across all layers of the CA1 area. In the control group, the number of GFAP and IBA-1-positive cells in the CA1 region increased significantly; the cell bodies were enlarged, and the processes were short and thick. In contrast, the number of GFAP and IBA-1-positive cells in the CA1 region of the SAHA group was significantly greater than in the control group. Conversely, the number of GFAP and IBA-1-positive cells in the ITSA group was significantly reduced compared to the control group.

### 4.7. ITSA-1 Reduces Histone Acetylation, Inhibits the Activation of the NF-*κ*B Pathway, and Supports Antineuronal Apoptosis

**Figure 5:**
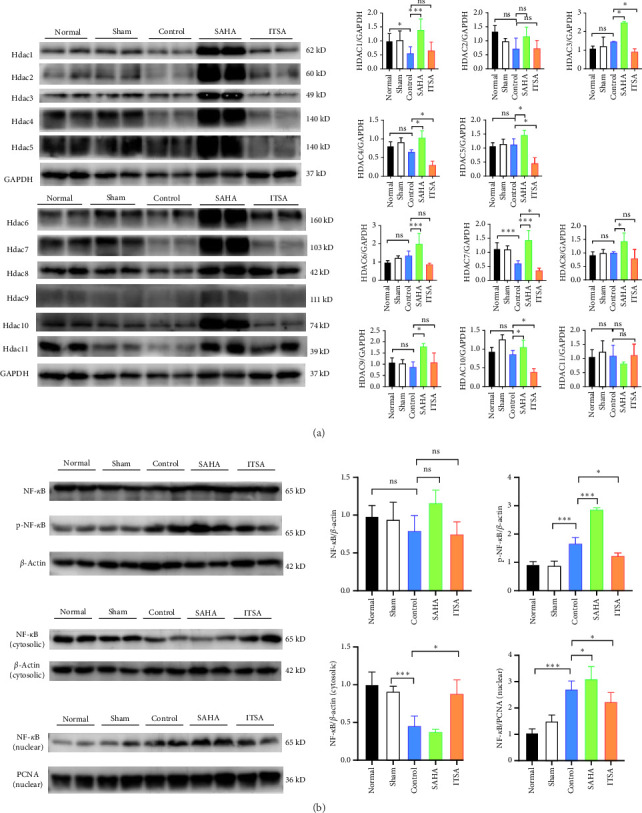
TSA-1 Reduces Histone Acetylation, Inhibits the Activation of the NF-*κ*B Pathway, and Supports Anti-Neuronal Apoptosis. The HDACs and NF-*κ*B proteins expression in brain hippocampal tissues after cardiac arrest and resuscitation was determined by Western blotting. (A) HDAC1-11 proteins expression in the rat hippocampus after ROSC, *⁣*^*∗*^*p* < 0.05; *⁣*^*∗∗∗*^*p* < 0.01; versus control group. (B) Western blotting analysis of NF-*κ*B P65 levels in the cytoplasm and nuclei of rat hippocampus after ROSC in different groups. *⁣*^*∗*^*p* < 0.05; *⁣*^*∗∗∗*^*p* < 0.01; versus control group. *N* = 3 rat per group.

Compared to the control group, SAHA increased histone acetylation in the rat hippocampus after ROSC, whereas ITSA decreased the level of histone acetylation. After ROSC, the expression of HDACs protein in the rat hippocampus decreased; however, SAHA treatment led to an increase in the expression of HDACs proteins. Although SAHA acts as a broad-spectrum HDACs inhibitor, the inhibition of HDACs enzyme activity results in feedback-induced upregulation of HDACs protein expression. In contrast, ITSA-1, acting as an activator of HDACs protease, induced the downregulation of HDACs protein expression. The protein levels of HDAC 3, 4, 5, 7, and 10 in the ITSA group were significantly lower than those in the control and SAHA group (Figure 5A).

After ROSC, the expression levels of HDACs genes decreased. While SAHA promoted the expression of HDACs genes, ITSA-1 inhibited HDACs gene expression.

The protein levels of phosphorylated NF-*κ*B and p65 in the nucleus were significantly increased in the rat hippocampus after ROSC. Compared to the control group, the protein levels of phosphorylated NF-*κ*B and p65 were significantly higher in the SAHA group, whereas these levels in the ITSA group were significantly lower than in both the control and SAHA groups (Figure 5B).

Compared to the normal and sham groups, the levels of caspase-3, cleaved caspase-3, and Bax protein in the hippocampus of rats in the control group after ROSC were significantly increased, while the levels of Bcl-2 protein were significantly decreased.

**Figure 6:**
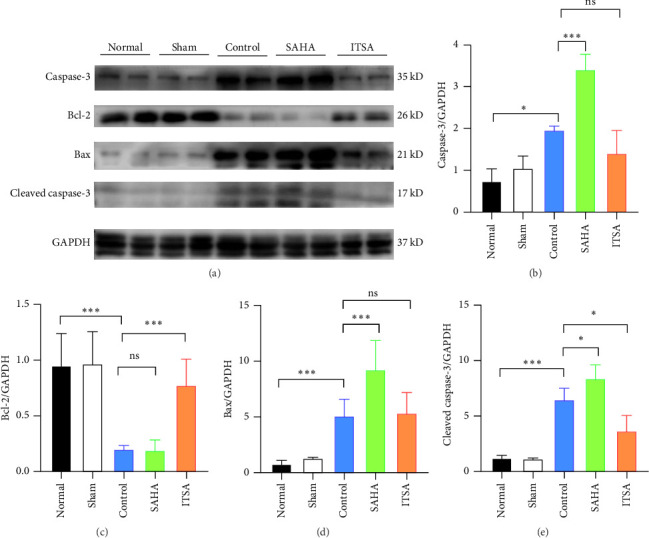
ITSA reduces the expressions of Caspase3, Bax, Bcl-2 and Cleaved caspase3 in the hippocampus of rats after cardiac arrest. (A) Different proteins expression in the rat hippocampus after ROSC. (B–E) The expressions of Caspase3, Bax, Bcl-2 and Cleaved caspase3. *N* = 3 rat per group. *⁣*^*∗*^*p* < 0.05; *⁣*^*∗∗∗*^*p* < 0.01.

Compared to the control group, the protein levels of caspase-3, cleaved caspase-3, and Bax in the SAHA group were significantly increased, and the levels of Bcl-2 were significantly decreased. In contrast, compared to the rats in the control and SAHA groups, ITSA-1 significantly decreased the levels of caspase-3, cleaved caspase-3, and Bax protein in the hippocampus of rats after ROSC and increased the level of Bcl-2 protein (Figure 6).

## 5. Discussion

In this study, asphyxia was used to induce CA in rats. After CA, the heart rhythms of the rats displayed a sequential transition from PEA to asystole, which more accurately simulates the pathophysiological processes of CA caused by nonshockable rhythms. Upon successfully establishing the CA model, we investigated the effects of the HDACs inhibitor SAHA and the activator ITSA-1 on heart and brain injury in rats following ROSC. Previous studies have employed varying standards for MAP, ranging from 10 to 30 mmHg, and the duration of CA has also differed, ranging from 5 to 12 min. Although different MAP thresholds and CA durations may have some impact on the extent of injury, in this study, Wistar rats demonstrated greater tolerance to hypoxia compared to other species. We strictly controlled the experimental conditions and applied a uniform standard of 30 mmHg. As a result, the degree of injury was largely consistent across all experimental subjects. Therefore, the selection of this standard is unlikely to have a significant effect on the overall trends, intergroup differences, or the conclusions drawn from our experimental results.

Study on the effects of ITSA-1 on brain injury and inflammatory factor expression following CA is currently in its nascent stages, with limited literature documentation. This study found that the application of ITSA-1 shortened the BLS duration, reduced the dosage of adrenaline needed during CPR, and decreased the levels of IL-1*β*, TNF-*α*, GFAP, and S100*β* in serum, as well as TNF-*α* and IL-1*β* in the hippocampus after ROSC for 4 h. Consequently, this improved cardiac and neurological function and the survival status of rats after ROSC. Further research revealed that ITSA-1 inhibited the activation of glial cells in the rat hippocampal CA1 region, reduced hippocampal histone acetylation, inhibited the activation of the NF-*κ*B pathway, and thus promoted the survival of neurons in the CA1 region of rats after ROSC. Conversely, SAHA had detrimental effects on rats after ROSC. Almost all rats in the SAHA group died from cardiogenic shock within 24 h after ROSC. The levels of IL-1*β*, TNF-*α*, GFAP, S100*β* in serum, and TNF-*α*, IL-1*β*, the activation of glial cells, and the NF-*κ*B pathway in the hippocampus of rats in the SAHA group after ROSC for 4 h were significantly higher than those in the control and ITSA groups. Plasma IL-1*β* and TNF-*α* levels after ROSC in the SAHA group were significantly higher than in the control group, indicating that SAHA can exacerbate postresuscitation syndrome, resulting in poor prognosis in rats. Conversely, the use of ITSA-1 before CA can alleviate postresuscitation syndrome, thus improving the cardiac and neurological functions of rats after ROSC. In addition to reducing the systemic inflammatory response after ROSC, ITSA-1 also reduced the activation of astrocytes and microglia in the CA1 region of the hippocampus, decreased IL-1*β* and TNF-*α* levels in the hippocampus, inhibited NF-*κ*B pathway activation, and promoted the survival of neurons in the CA1 region of rats after ROSC. While long-term follow-up is equally important, this study concentrates on the critical early stage, laying the foundation for subsequent in-depth research. Through experimental research, we discovered that the ITSA demonstrates significant neuroprotective and cardiovascular protective effects during the early stage of CA and CPR in rats.

The findings of this study appear to diverge from previous research investigating local organ ischemia-reperfusion injury, presenting potential discrepancies in mechanistic interpretations [20–23]. This discrepancy may be related to the different models used in the study; their studies focused on ischemia-reperfusion injury in isolated single organs, in contrast to our comprehensive investigation of systemic inflammatory responses across multiple organ systems. In our research, asphyxia was used to induce CA; the CA lasted for 12 min, resulting in severe whole-body hypoxia, and the subsequent CPR and ROSC caused reperfusion injury. The symptoms of postresuscitation syndrome in rats after ROSC were severe, with significant increases in plasma inflammatory factors IL-1*β* and TNF-*α* in the control group and impaired cardiac and neurological functions. Studies have shown that reducing the systemic inflammatory response after ROSC may improve the prognosis of patients with CA. The use of steroid hormones before or after the initiation of CPR decreased plasma IL-6 and TNF-*α* levels after ROSC, significantly increased the likelihood of ROSC, and improved survival to hospital discharge with favorable neurological status [24, 25].

By investigating the relationship between the NF*κ*B pathway and HDAC-dependent transcriptional regulation, we discovered a critical molecular mechanism in inflammatory responses. NF-*κ*B is a central transcription factor whose expression pattern and subsequent role depend greatly on the type and manner of cerebral injury. Blocking the NF-*κ*B-mediated inflammatory response may play a protective role in postresuscitation brain injury and myocardial dysfunction [26–28]. Acetylation plays a prominent role in regulating the nuclear action of NF-*κ*B. The RelA subunit of NF-*κ*B, the major target of acetylation, is modified at several lysine residues. Acetylation of discrete lysine residues in RelA modulates distinct functions of NF-*κ*B, including transcriptional activation, DNA binding, and assembly with its inhibitor I*κ*B*α* [29, 30]. Studies have suggested that HDAC3 inhibition activates inflammatory cytokine signaling through cartilage degradation, thereby increasing histone acetylation in chromatin [31]. HDAC 4 inhibits NF-*κ*B activation by facilitating I*κ*B*α* sumoylation [32, 33]. One overall role of HDACs is to inhibit NF-*κ*B activation through molecular mechanisms specific to the stimulus and promoter [34].

NF*κ*B precisely regulates inflammatory gene expression through interactions with HDAC enzymes, modulating cellular stress and immune functions. This complex molecular regulatory network provides an important theoretical basis for developing inflammatory intervention strategies after CA, offering new therapeutic approaches for neuroprotection.

Studies indicated that HDAC inhibitors may cause adverse cardiac effects, including reduced myocardial electrical activity and prolonged myocardial cell repolarization, making the heart more susceptible to potentially lethal arrhythmias [35, 36]. This study observed that animals in the SAHA group experienced earlier occurrences of PEA and asystole, while the use of ITSA-1 shortened the duration of PEA and asystole, thereby increasing the likelihood of achieving ROSC in rats after CA. Research indicates that HDAC may be used to treat supraventricular arrhythmia, myocardial infarction, cardiac remodeling, hypertension, and fibrosis. Despite these preliminary insights, the existing research evidence remains limited, underscoring the imperative for further in vivo and clinical studies to elucidate the precise molecular mechanisms. Based on our findings, we hypothesize that the suppression of systemic inflammatory responses may be closely linked to the cardiac electrophysiological activity and controlled local inflammatory reactions within the brain. This potential correlation suggests a sophisticated inflammatory regulation mechanism where modulating systemic inflammation could potentially influence and mitigate localized neuro-inflammatory processes, thereby providing a novel perspective on neurological protection strategies following CA.

The results of the present study showed that ITSA-1 activates the activity of HDACs 3, 4, 5, 7, and 10, as verified by Western blot, suggesting that HDACs are crucial for inhibiting the activation of the NF-*κ*B pathway and regulating pro-inflammatory gene expression. This potential observation may implicate off-target effects, warranting more rigorous and in-depth investigations in future research endeavors.

To date, no studies have confirmed that the HDAC activator ITSA-1 provides protection to organs in ischemia-reperfusion injury. This study is the first to confirm that activating HDACs activity with ITSA-1, reducing histone acetylation, and inhibiting the activation of the NF-*κ*B pathway can reduce the systemic inflammatory response after CA-ROSC and alleviate cardiac and neurological impairments in rats after ROSC. It may related to increased HDAC protease activity, promotion of histone deacetylation, and inhibition of the inflammatory response after ROSC. The specific mechanisms, however, still require further investigation for clarification. This study has several limitations. First, we did not conduct in vitro cellular experiments to assess the effects of SAHA and ITSA-1 on the survival of cardiomyocytes and neuronal cells following ischemia-reperfusion injury. Second, we did not evaluate the specific targets through which SAHA and ITSA-1 exert their effects, which necessitates further research for confirmation. The exclusive use of male rats limits the generalizability of our findings. Given known sex differences in inflammatory responses and cardiac function, future studies may investigate potential sex-specific effects of ITSA-1 treatment and optimization of drug dosing, strategies, potential side effects, and combination therapies. In this study, the drug was administered prophylactically to enhance experimental controllability and ensure the survival and stability of experimental subjects. Compared to therapeutic administration, it may reduce the clinical relevance, thereby potentially compromising the guiding significance of research findings. Furthermore, in this study, CA was defined as a MAP below 30 mmHg rather than 20 mmHg, which is inconsistent with the standards used in some other experimental animal models. This represents a limitation of our study, as different MAP thresholds may have varying impacts on the extent of injury. However, there is currently no universally accepted standard for this parameter in the scientific community. Therefore, we plan to further refine our model parameters in future experimental designs to enhance the generalizability and comparability of our research findings.

## 6. Conclusion

The HDAC activator ITSA-1 improves the outcomes of heart and brain injuries in rats and increases survival rates after ROSC by reducing histone acetylation and alleviating systemic inflammatory responses. Its mechanism may involve inhibiting the activation of glial cells in the brain, increasing the survival of neurons in the brain tissue of rats after ROSC, and enhancing CO and EF. This provides a new therapeutic strategy for the prevention and treatment of cardiac and brain injuries following CA and CPR.

## Figures and Tables

**Table 1:** The physiological parameters and CPR parameters of rats in each group.

Group	Normal	Sham	Control	SAHA	ITSA
*n*	8	8	15	15	15
BW (g)	379.60 ± 23.17	377.80 ± 21.48	380.20 ± 13.83	385.10 ± 11.39	385.10 ± 20.39
HR (beats/min)	384.42 ± 28.54	377.85 ± 19.50	388.57 ± 30.01	368.14 ± 21.77	391.42 ± 31.53
SP (mmHg)	143.4 ± 14.43	140.2 ± 13.36	144.1 ± 15.13	138.4 ± 6.43	137.4 ± 15.28
DP (mmHg)	129.6 ± 14.70	129.6 ± 12.64	133.8 ± 13.63	126.4 ± 5.52	119.8 ± 13.80
MAP (mmHg)	136.8 ± 13.73	136 ± 12.74	139.9 ± 14.30	132.8 ± 6.01	129.4 ± 12.85
PEA (s)	0	0	295.2 ± 38.36	301.9 ± 33.85	263.5 ± 32.49^*∗*^^#^
p-Asystole (s)	0	0	70.90 ± 35.38	197.40 ± 71.02^*∗∗∗*^	54.00 ± 40.06^*∗∗∗*^^###^
BLS time (s)	0	0	318.70 ± 231.40	307.90 ± 204.30	145.90 ± 87.15^*∗*^^#^
Ventricular fibrillation (%)	0	0	0	0	0
Adrenaline dosage (µg)	0	0	0.72 ± 0.41	0.76 ± 0.35	0.52 ± 0.19^*∗*^^#^

*Note:* Data were expressed as mean ± standard deviation (SD). The comparison of means between two samples was conducted using the *t*-test, while comparisons among data from more than three groups were performed using one-way analysis of variance (ANOVA). Eight rats were excluded from the experiment due to complications.

Abbreviations: BLS, base life support; BW, body weight; DP, diastolic pressure; HR, heart rate; MAP, mean arterial pressure; PEA, pulseless electrical activity; SP, systolic pressure.

*⁣* ^*∗*^Compared with control group, *p* < 0.05.

*⁣* ^*∗∗∗*^Compared with control group, *p* < 0.001.

^#^Compared with SAHA group, *p* < 0.05.

^###^Compared with SAHA group, *p* < 0.001.

## Data Availability

The datasets used and/or analyzed during the current study are available from the corresponding author upon reasonable request.
